# Supplementary material for: Attenuation of Ventilation-Induced Endoplasmic Reticulum Stress Associated with Lung Injury Through Phosphoinositide 3-Kinase-Gamma in a Murine Endotoxemia Model
Source: Int J Mol Sci. 2025 Jun 16;26(12):5761. doi: 10.3390/ijms26125761 (PMC12192643; doi:10.3390/ijms26125761)
Supplement: Supplementary file 1 [file ijms-26-05761-s001.zip › ijms-3598788-supplementary.pdf]

**The following data are supplementary materials.**

Epithelial apoptosis was characterized by nuclear condensation of the bronchial epithelium in the mice treated with MV and endotoxin (Fig. S1). Apoptosis in the airway epithelia stimulated by treatment with MV and endotoxin were reduced in PI3K-γ-deficient mice than in control mice ($p < 0.05$; Fig. 6C).

**Figure S1.** PI3K-γ homozygous knockout ameliorated lung stretch-induced epithelial apoptosis. Representative micrographs with TUNEL staining of paraffin lung sections and quantitation (X400) were from the lungs of nonventilated control mice and mice ventilated with a tidal volume ($V_T$) of 30 mL/kg for 5 h with or without LPS administration (n = 5 per group). A dark-brown diaminobenzidine signal indicated positive staining of apoptotic cells, whereas shades of blue–green to greenish tan signified nonreactive cells. Apoptotic cells are identified by arrows. Scale bars represent 20 nm. TUNEL = terminal deoxynucleotidyl transferase-mediated dUTP-biotin nick end-labeling; LPS = lipopolysaccharide; 4-PBA = 4-phenylbutyric acid; PI3K-γ = phosphoinositide 3-kinase-γ; VT = tidal volume.

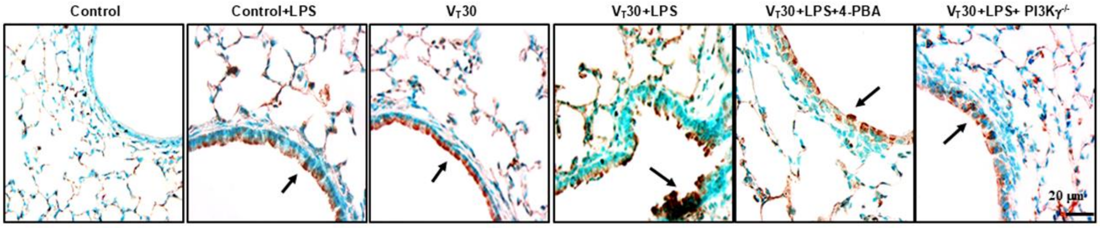

**Table S1.** Physiologic conditions at the beginning and end of ventilation.

| | Nonventilated | Nonventilated LPS | $V_T$ 30 ml/kg | $V_T$ 30 ml/kg LPS | $V_T$ 30 ml/kg LPS+ 4-PBA | $V_T$ 30 ml/kg LPS+PI3Kγ$^{-/-}$ |
|---|---|---|---|---|---|---|
| PH | 7.41±0.04 | 7.38±0.02 | 7.36±0.07 | 7.37±0.04 | 7.37±0.06 | 7.39±0.04 |
| PaO2 (mmHg) | 98.5±0.2 | 93.1±0.2 | 79.6±2.2* | 75.1±2.8* | 85.6±2.4* | 87.3±1.8* |
| PaCO2 (mmHg) | 39.1±0.3 | 39.6±0.3 | 38.4±1.6 | 38.5±1.4 | 37.8±1.2 | 37.6±1.5 |
| MAP (mmHg) | | | | | | |
| Start | 85.6±1.2 | 83.7±0.5 | 82.7±2.5 | 82.4±2.7 | 84.7±2.1 | 84.6±2.4 |
| End | 85.1±0.4 | 81.2±0.4 | 78.7±1.8* | 74.9±2.1* | 78.2±2.3* | 78.5±2.4* |
| PIP (mmHg) | | | | | | |
| Start | | | 16.3±1.2 | 16.1±1.5 | 15.9±1.4 | 15.8±1.2 |
| End | | | 17.4±1.8 | 17.6±1.8 | 17.2±1.3 | 17.0±1.5 |

At the end of the study period, we obtained data of mean arterial pressure and arterial blood gases from the nonventilated control mice and mice ventilated at a tidal volume of 30 ml/kg for 5 h (n = 10 per group). The normovolemic statuses of mice were maintained by monitoring mean artery pressure. Data are presented as means ± SDs. * Indicates that $P < 0.05$ when compared to the nonventilated control mice with LPS. LPS = lipopolysaccharide; MAP = mean arterial pressure; 4-PBA = 4-phenylbutyric acid; PI3Kγ-/- = phosphoinositide 3-kinase-γ-deficient mice; PIP = peak inspiratory pressure; VT = tidal volume.
